# Challenges and lessons from a primary care intervention in a Brazilian municipality

**DOI:** 10.11606/S1518-8787.2019053000457

**Published:** 2019-04-26

**Authors:** Mônica V Andrade, Kenya Noronha, Clareci S Cardoso, Claudia D L Oliveira, Júlia A Calazans, Michelle N Souza

**Affiliations:** I Universidade Federal de Minas Gerais. Departamento de Economia. Centro de Desenvolvimento e Planejamento Regional. Belo Horizonte, MG, Brasil; II Universidade Federal de São João del-Rei. Campus Centro-Oeste. Departamento de Saúde Coletiva. Divinópolis, MG, Brasil; III Universidade Federal de Minas Gerais. Departamento de Demografia. Centro de Desenvolvimento e Planejamento Regional. Belo Horizonte, MG, Brasil

**Keywords:** Chronic Disease, prevention & control, Community Health Workers, Community Health Services, Health Care Levels

## Abstract

**OBJECTIVE:**

To address the implementation of the Lab for Innovation in Chronic Conditions in Santo Antonio do Monte, indicating the main challenges and lessons of a new chronic condition model.

**METHODS:**

This is an observational study based on two sources of data: 1) two cross-sectional household surveys, 2013 (2012 as reference year) and 2015 (2014 as reference year), representative for the entire population and four target groups (pregnant women; children under two years old; individuals with hypertension and diabetes); medical records of individuals who self-reported having hypertension or diabetes in the household survey of 2013. A descriptive statistics analysis was performed.

**RESULTS:**

The main findings showed that the public health system is the main provider of health services, mainly primary care, in Santo Antonio do Monte. Besides, the implementation of Lab for Innovation in Chronic Conditions showed the importance of building a Primary Health Care network in small municipalities.

**CONCLUSIONS:**

Community health agents and health managers played a fundamental role in the Primary Health Care network. The case study of Santo Antonio do Monte poses some challenges and lessons that clarify future interventions on building a Primary Health Care network that is essential to provide an adequate and longitudinal care to chronic conditions.

## INTRODUCTION

After the Brazilian Unified Health System (SUS) was created, in 1988, several policies have been implemented to consolidate a universal health coverage in Brazil^1–3^. SUS is a public decentralized system in which municipalities and states manage and provide health services. Given their knowledge about the population’s needs, their main role is to organize primary health care (PHC).

In 2010, Brazil had 5,565 municipalities, 70% of them had less than 20,000 inhabitants^4^. The small population size associated with low levels of gross domestic product *per capita* (GDP) raises challenges for the organization and delivery of PHC at the municipal level. In 1994, the federal government implemented the Family Health Strategy (FHS)^2^ to consolidate PHC. FHS is a geographically and community-based program that strengthens the relationship between providers and patients. It represented an important change in the public healthcare model when it shifted from a curative to a preventive care^5–7^. According to this strategy, families are the focus of public health policies that should cover primary care for all population groups. The Family Health Teams (FHT) are composed of a family physician, a nurse, a nursing assistant and at least 4–12 community health agents (CHA). CHA are recruited because they can easily identify the local needs of the population. They must be able to detect symptoms of diseases, to refer individuals to the care needed and identify the need for continued care, acting as the bridge between community and public health system^8–10^. Empirical evidence has already been raised about the recent improvements observed in the population health status and access to healthcare due to FHS coverage^3,8,11–14^.

In Brazil, the burden of chronic conditions due to the aging process and unhealthy life-style has increased^15^. It is noteworthy that chronic conditions refer to a broader concept than only the presence of chronic diseases. They can be defined as health conditions that persist over time and require ongoing management. This approach classifies persistent communicable diseases (e.g. HIV/AIDS and tuberculosis), mental disorders and impairments as chronic conditions^16^. Mendes^17^ has expanded this concept by considering maternity and perinatal health care needs related to life cycles, such as child, adolescent and elderly care. Therefore, the creation of a network that includes primary, secondary and tertiary health services is fundamental to provide an integral and longitudinal healthcare. The traditional model targeted to acute conditions is not able to sustain satisfactory health outcomes when they have to deal with chronic conditions^18^.

The Brazilian Ministry of Health has already recognized these caveats and launched SUS Healthcare Network Policy in 2010. Within this scope, the Lab for Innovation in Chronic Conditions (LIACC) is a new management tool of PHC based on the Attention to Chronic Conditions Model (ACCM) adapted by Mendes^17^ to be applied in the Brazilian context. The tool was implemented in three cities: Curitiba (2011), Santo Antônio do Monte (2013) and Tauá (2014). The aim of this study is to address the implementation of LIACC in Santo Antônio do Monte (SAMONTE), as well as the main challenges faced by the municipality in the provision of this new chronic condition model. SAMONTE experience was a population-based intervention that involved all providers of primary and secondary care.

Santo Antônio do Monte is a small municipality with around 26,000 inhabitants and located in Minas Gerais, Brazil. It is a high-developed municipality presenting a Municipal Human Development Index of 0.724 in 2010 and infant mortality of 14.8 deaths per 1,000 live births^4^. Most of its population lives in the urban area (85%), in households provided with good sanitation conditions^4^. In 2012, 92% of its population was covered by eight FHT. Health care supply also included a secondary care facility specialized in care for chronic conditions (*Centro Integrado Hiperdia Viva Vida*), a specialist dental center, an image diagnostic center, a small hospital (50 beds), and a clinical analysis laboratory.

This study addresses at least two main questions related to the LIACC intervention: what lessons can we learn from this experience and what are the main challenges that should be overcome for a more suitable chronic care model at the municipal level? We believe that the SAMONTE experience can be an important opportunity for other municipalities with similar population size and development level.

## METHODS

### LIACC in Santo Antônio do Monte

The conceptual framework of LIACC is the ACCM adapted to the Brazilian context by Mendes^17^. ACCM includes components of the Chronic Care Model^19^, the Kaiser Pyramid Model^20^ and the Social Model of Health^21^. ACCM proposes seven steps to organize the macroprocesses of PHC (Box 1). LIACC was implemented in SAMONTE as a partnership among the Pan-American Health Organization, the Government of the State of Minas Gerais, the Government of Santo Antônio do Monte, and the National Council of Health Secretaries (CONASS). The information about the LIACC implementation was collected through several technical meetings with Pan-American Health Organization consultants and local staff of SAMONTE who were responsible for the intervention. In addition, we had access to all material used in the workshops to introduce the new tools of primary care to local providers.

**Box 1:** The seven steps of the chronic conditions model care (ACCM) and their main macroprocesses.

Steps	Main macroprocesses
1. Evaluation of the existing infrastructure and definition of improvements needed considering the reference population	Territorialization, registry of families, family risk classification, risk stratification of chronic conditions.
2. Primary care to acute health events	Patients’ reception at Health Unit, Manchester triage system, delivery of primary care according to Manchester protocol.
3. Primary care to patients with controlled chronic conditions	Management and monitoring of chronic conditions, risk stratification of chronic conditions, elaboration of patients care plan, self-care support.
4. Preventive care and tools to support behavior change	Physical activities, control of alcohol, tobacco and drugs, and nutritional re-education groups, motivational interviewing.
5. Administrative demands	Control of medical certificates, renewal of prescription of drugs and medicines, management of Health Units including information system, medical records and public health surveillance.
6. Home healthcare	Management and monitoring of household visits.
7. Self-care support	Self-care training and advice, elaboration of patients care plan, material support to self-care.

Source: Mendes^17^.

The intervention occurred in the municipality between June 2013 and December 2014. It focused on four chronic conditions (pregnant women; children under two years old; individuals with hypertension and diabetes) involving all health professionals of primary and secondary care. This intervention aims to strengthen PHC for chronic conditions by using new tools and protocols developed to manage a longitudinal and integrated network.

The development of LIACC was based on the introduction of the macroprocesses in workshops and a tutoring program delivered by health policy specialists. In each workshop, a schedule of activities and goals were defined for the Health Units (HU) using standard procedures. The tutors developed a set of protocols and clinical guidelines to be followed by PHC providers. A health manager was hired for each HU to guarantee the success of these strategies. They were responsible for articulating all actors of HU, monitoring the use of protocols and clinical guidelines, and managing the process by conducting weekly meetings with FHT.

Steps 1, 2, 3 and 6 in Box 1 were implemented in all HU, while technologies related to the remaining steps were not systematically introduced. The introduction of these steps were conditioned to the ability of health professionals to understand each macroprocess and their engagement with the LIACC goals. Regarding the information system, an electronic record system should be implemented integrating HU and secondary care providers. However, the municipality did not succeed in reaching this goal.

These innovations were introduced mainly in HU, but also involved the secondary care facility, which allowed the integration of these two health care levels. The macroprocess of territorialization, registry of families, family risk classification, risk stratification of chronic conditions, and the use of the Manchester protocol after a deep change in the organization of PHC. FHT became more aware of the risk and vulnerable conditions of its population, increasing its ability to provide the care needed and reducing unnecessary referrals to secondary care facility. Therefore, the flow of patients between different levels of attention became more coordinated. It is noteworthy that the secondary care facility has already played an important role in providing outpatient care for high-risk patients diagnosed with hypertension, diabetes and women with high-risk pregnancy. It also has provided care to patients living in 12 neighbouring municipalities of SAMONTE. The presence of *Centro Hiperdia Viva Vida* and its regional importance motivated the choice of SAMONTE for receiving the intervention.

### Study Design

This is an observational case study based on two sources of data. The first source comprises two cross-sectional household surveys conducted in 2013 and 2015. The reference period for each survey was 2012 and 2014, before and during the intervention, respectively. All information was self-reported except for child immunization. In this case, a picture of the child’s immunizations card was taken during the interview.

The surveys are representative for the entire population and the target groups: pregnant women; children under two years old; individuals with hypertension and diabetes.

The sample of the entire population is probabilistic at the census sectors level and representative for the population aged between 18 and 65 years with a margin of error equal to 4%. The sample is stratified by HU. Individuals were selected on the basis of quota sampling by age and sex. In total, 596 individuals were interviewed in 2013 and 625 in 2015. Information about household characteristics, socioeconomic status, health status, lifestyle, and health care utilization was collected.

Pregnant women included those who had completed their pregnancy with a live birth between January 2011 and December 2012 (first round of the household survey) and between January 2014 and December 2014 (second round of the household survey). The final sample consisted of 336 pregnant women and their respectively children (183 in 2013 and 155 in 2015), 744 individuals with hypertension (365 in 2013 and 379 in 2015), and 629 individuals with diabetes (312 in 2013 and 317 in 2015). Specific instruments were defined for each target group including information about health status and health care access. Individuals of each target group also answered all questions of the instrument applied to the entire population. The distribution of age and sex for each target group are very similar for both household surveys, which guarantee comparability between both years^22^.

Two groups of outcome variables were selected (Box 2). The first one consists of indicators closely correlated to some macroprocesses of ACCM. The analysis was performed for the macroprocesses that were implemented in SAMONTE and able to be measured by the available sources. The second group includes health outcomes associated with PHC. All indicators are dummy variables equal to one if the attribute was observed, zero otherwise. The only exception is the type of provider for antenatal care (ANC), whose variable has three categories: FHS, other professionals of SUS and private provider. A descriptive statistics analysis was performed based on a comparison of two cross-sections carried out in 2013 and 2015.

**Box 2:** Health indicators related to macroprocesses of the ACCM model.

Variable	Definition	Data source
Health indicators related to macroprocesses		
Familiar risk classification	Family risk classification considering social vulnerability, familiar and individual risk factors	Medical records
Risk stratification	Risk stratification of patients with diabetes or hypertension	Medical records
Registry of families	Enrolment status of the households by FHT	Household survey
CHA household visits	Household was visited by CHA at least once in the last 12 months	Household survey
SUS users	Individual usually receive care provided by SUS	Household survey
Private health insurance coverage	Individual has private health insurance coverage	Household survey
Diabetes	Individual self-reported having diabetes	Household survey
Hypertension	Individual self-reported having hypertension	Household survey
Health outcomes for Individuals with hypertension and diabetes		
Doctor visit	Individual visited a doctor at least once in the last 12 months	Household survey
Doctor visit by SUS	Individual visited a doctor of SUS at least once in the last 12 months	Household survey
ECG test	Individual received at least one electrocardiogram test in the last 12 months	Household survey
Blood test or cholesterol test	Individual with diabetes did at least one blood test or Individual with hypertension did at one cholesterol test in the last 12 months	Household survey
Health outcomes associated with antenatal care (ANC), delivery, and child care		
ANC provider	Main provider of ANC care: FHT, other professionals of SUS, private	Household survey
At least 6 ANC visits	Woman received at least 6 ANC visits during pregnancy	Household survey
Early prenatal care	Receiving care during the first three months of pregnancy	Household survey
Antenatal care tests	Tests performed during pregnancy: blood test, toxoplasmosis test, HIV and syphilis test, urine test, oral glucose test, ultrasound test	Household survey
Immunization	Woman was immunized against tetanus and influenza during pregnancy	Household survey
Preterm birth	Baby birth before 37 weeks of gestational age	Household survey
Vaginal delivery	Baby was born through vaginal delivery	Household survey
C-section scheduled	C-section scheduled during the ANC period	Household survey
Child immunization	Child immunization against BCG, hepatitis, poliomyelitis, DTP, rotavirus, meningitis, MMR	Household survey
Low birth weight	Birth weight below 2,500 kg	Household survey

Source: SAMONTE^24^.

FHT: family health teams; CHA: community health agent; SUS: Brazilian Unified Health System; ECG: electrocardiogram; BCG: vaccine against tuberculosis; DTP: vaccine against diphtheria, pertussis, and tetanus; MMR: vaccine against measles, mumps, and rubella

This study meets all ethical requirements involving research with humans established by the 196/96 Resolution of the Ministry of Health. The protocol was submitted and approved by the Research Ethics Committee at the Universidade Federal de São João Del Rei (Process CEP 369.942). The Informed Consent form was read and signed by all interviewees, and a copy was properly filed.

The second source of data comprises medical records of all individuals interviewed in 2013 who had been diagnosed with hypertension or diabetes. We collected information in all HU of SAMONTE for 2012, 2013 and 2014. In this study, three indicators were analyzed: the percentage of medical records identified in the HU, individual risk stratification and family risk classification.

## RESULTS

Table 1 shows the findings for the entire population, individuals with diabetes and hypertension. In 2012, 95.88% of the entire population reported that the household was registered in FHS, while 90.94% reported having received at least one CHA visit during the reference year. In 2014, these percentages were 93.65% and 95%, respectively. The increase in the coverage of CHA visit between the two years was statistically significant, while the reduction in the FHS coverage was not significant. Besides, more than 90% of the individuals reported having used public health services. The prevalence of individuals who declared having hypertension remained almost constant over time (26.55% in 2012 and 27.72% in 2014), but a significant increase was found in the proportion of those who had diabetes, 4.53% to 9.44%.

**Table 1:** Health indicators for the entire population and individuals with hypertension and diabetes before (2012) and during (2014) the LIACC intervention in Santo Antônio do Monte, Brazil.

Variable	Entire population						Diabetes						Hypertension					
		
	2012		2014		χ^2^ test (p-value)		2012		2014		χ^2^ test (p-value)		2012		2014		χ^2^ test (p-value)	
					
	n	%	n	%			n	%	n	%			n	%	n	%		
Household survey																		
Registry of families in FHS	558	95.88	575	93.65	0.085	NS	305	98.71	308	97.47	0.260	NS	350	97.77	364	96.55	0.324	NS
CHA household visits	532	90.94	588	94.99	0.006	^a^	294	94.53	299	94.62	0.962	NS	327	90.08	359	94.97	0.011	^b^
SUS patients	554	92.95	571	91.36	0.301	NS	310	99.36	309	97.78	0.927	NS	360	98.63	364	96.04	0.029	^b^
Private health insurance	184	30.87	190	30.50	0.887	NS	107	34.41	108	34.07	0.929	NS	142	38.90	146	38.52	0.915	NS
Diabetes prevalence	27	4.53	59	9.44	0.001	^a^												
Hypertension prevalence	158	26.55	173	27.72	0.646	NS												
Doctor visit							291	95.10	278	89.39	0.008	^a^	303	85.11	305	81.12	0.150	NS
Doctor visit by SUS (conditional)							216	74.23	219	78.78	0.201	NS	207	68.54	199	65.25	0.3890	NS
ECG test							185	61.67	195	62.30	0.872	NS	174	48.07	173	45.77	0.531	NS
Blood test							276	92.00	270	89.40	0.273	NS						
Cholesterol test													259	71.35	281	74.73	0.300	NS

Source: SAMONTE^24^.

LIACC: Lab for Innovation in Chronic Conditions; FHT: family health teams; CHA: community health agent; SUS: Brazilian Unified Health System; ECG: electrocardiogram; NS: not significant;

^a^ Statistically significant at 1%.

^b^ Statistically significant at 5%.

Among individuals with diabetes, the FHS coverage is also universal, around 98%. Additionally, the percentage of those using SUS services and receiving at least one CHA visit in the reference year is quite high, around 98% and 95%, respectively, remaining stable between both years.

The percentage of individuals with diabetes who visited a doctor during the reference year significantly decreased between 2012 and 2014, from 95.10% to 89.39%. Among them, 74.23 and 78.78% received this care through SUS in 2012 and 2014, respectively. Regarding preventive tests, a reduction was found in the coverage of blood test between 2012 and 2014, from 92% to 89.4%, but it was not significant. The coverage of ECG test remained stable, around 62%.

As for individuals with hypertension, the FHS coverage was also almost universal, around 97% in both years. Moreover, the percentage of those who received at least one CHA visit in the reference year increased significantly, from 90.08% in 2012 to 94.97% in 2014. In contrast, the percentage of those who declared regular use of SUS services decreased from 98.63% in 2012 to 96.04% in 2014. Despite this reduction, the use of public services is too high, especially considering that more than 35% of individuals with hypertension are covered by private health insurance.

More than 80% of individuals with hypertension reported receiving at least one doctor visit in both years, and more than 65% of them were treated by SUS. As for the preventive tests, the percentage of people who did at least one cholesterol test is not universal, but the coverage is high, around 75%. For ECG, this number was lower, around 46%. No statistically significant changes were found in the coverage of both tests between 2012 and 2014.

The analysis of medical records brought positive evidence for the macroprocesses implemented by LIACC. About 84% and 74% of individuals who self-reported having diabetes and hypertension were identified in their medical records at HU diagnosed with the respective condition, besides receiving treatment by FHS. Among them, 80% of individuals with diabetes and 89% with hypertension lived in households that had a medical history of family risk classification in their medical records. Most risk classifications were carried out with the LIACC implementation between 2013 and 2015 (71.4% and 64.7% of the family risk classification for diabetes and hypertension, respectively). The percentage of individuals with risk stratification record, despite being at low levels, ranges from zero to 35% and 28%, respectively (Table 2).

**Table 2:** Family risk classification and individual risk stratification according to the medical records of patients with diabetes and hypertension in SAMONTE.

Variable	Diabetes		Hypertension	
	
	n	%	n	%
Family risk classification in the medical records of the patient				
No	43	16.9	23	8.5
Yes	203	79.9	241	88.6
Does not know	8	3.2	8	2.9
Total	254	100	272	100
The most recent year of family risk classification				
2009	9	4.4	10	4.2
2010	3	1.5	2	0.8
2011	0	0.0	2	0.8
2012	2	1.0	2	0.8
2013	87	42.9	68	28.2
2014	52	25.6	69	28.6
2015	6	3.0	19	7.9
No record of the year	44	21.7	69	28.6
Total	203	100	241	100
Individual risk stratification in the medical records of the patient				
No	166	65.35	195	71.7
Yes	88	34.65	77	28.3
Total	254	100	272	100

Source: SAMONTE^24^.

Regarding ANC, the coverage is universal (results not shown here), and FHS performs an important role since more than 70% of women were monitored by this program during their pregnancy (Table 3). In addition, the quality of ANC seems to be satisfactory. Table 3 shows that most women had at least six antenatal visits during pregnancy (91.33% in 2012 and 96.64% in 2014) and received early prenatal care (85.16% in 2012 and 94.12% in 2014). The coverage of both indicators increased between the two years, and this improvement was statistically significant. The coverage of prenatal tests is also almost universal. The exception stands for oral glucose test, whose prevalence is still lower. However, its coverage significantly increased after the LIACC intervention, from 33.33% in 2012 to 60.78% in 2014. Regarding immunization coverage, no significant changes were observed during the period. In 2012, about 77.60% and 90.71% of pregnant women were immunized against influenza and tetanus, respectively. In 2014, these figures were 75.16% and 91.50%. Despite the outcomes observed for ANC, childbirth characteristics did not present a good performance. Only 21% of women had vaginal delivery. Besides, almost 11% had low-birth-weight infants and around 27% had a preterm birth. These indicators showed little variability over time, except for the pre-schedule C-section births that decreased from 65.28% to 50.82% (Table 3). Figures for child immunization showed universal coverage for all children aged between 0–2 years (Table 3).

**Table 3:** Antenatal care, delivery and child immunization according to vaccination card before (2012) and during (2014) the LIACC intervention in Santo Antônio do Monte, Brazil.

Variable	2012		2014		χ^2^ test (p-value)	
	
	n	%	n	%		
Antenatal care and delivery						

ANC provider						
FHT	130	72.63	114	75.00	0.132	NS
Other professionals of SUS	11	6.15	3	1.97		
Private	38	21.23	35	23.03		
At least 6 ANC visits, since she did the ANC	158	91.33	144	96.64	0.049	^b^
Early prenatal care, since she did the ANC	156	85.16	144	94.12	0.008	^a^
Antenatal care tests						
Blood test	180	98.36	153	100.00	0.112	NS
Toxoplasmosis test	150	81.97	138	90.20	0.061	NS
Urine test	178	97.27	153	100.00	0.039	^b^
HIV and syphilis test	167	91.26	146	95.42	0.284	NS
Oral glucose test	61	33.33	93	60.78	0.000	^a^
Ultrasound test	182	99.45	153	100.00	0.360	NS
Immunization						
Immunized against influenza	142	77.60	115	75.16	0.601	NS
Immunized against tetanus	166	90.71	140	91.50	0.765	NS
Preterm birth	48	26.23	42	27.45	0.527	NS
Vaginal delivery	39	21.31	31	20.26	0.813	NS
C-section scheduled, since the delivery was a c-section	94	65.28	62	50.82	0.017	^b^
Low birth weight	21	11.67	17	10.97	0.841	NS

Child immunization according to the vaccination card						

BCG	157	100.00	131	100.00		
Hepatitis B (HepB)	158	99.37	130	99.24	0.891	NS
Oral polio vaccine (OPV)	158	100.00	121	100.00		
DTP	159	100.00	120	99.17	0.278	NS
Rotavirus	157	99.37	120	100.00	0.372	NS
Pneumococcal	157	100.00	119	100.00		
Meningococcal	159	100.00	119	100.00		
MMR	129	98.47	23	100.00	0.244	NS
Yellow fever	137	100.00	27	100.00		

Source: SAMONTE^24^.

LIACC: Lab for Innovation in Chronic Conditions; ANC: antenatal care; FHT: family health teams; SUS: Brazilian Unified Health System; BCG: vaccine against tuberculosis; DTP: vaccine against diphtheria, pertussis, and tetanus; MMR: vaccine against measles, mumps, and rubella. The vaccine coverage was calculated based on the number of eligible children for each vaccine according to the children’s age; NS: not significant

^a^ Statistically significant at 1%.

^b^ Statistically significant at 5%.

## DISCUSSION

The case study of SAMONTE raises some challenges and lessons that clarify future interventions on building a PHC network. The first lesson is the importance of SUS as the main provider of primary care in small municipalities. According to our findings, the FHS coverage in SAMONTE was universal even considering the high coverage of private health insurance. The importance of FHS was already noticeable before the intervention, since almost 100% of the households were registered in FHS and 91% received CHA visits. These figures are even higher than the coverage observed in Brazil. In 2013, 63% of the households in the country were registered in FHS and about 83% declared receiving at least one CHA visit^23^.

The second lesson shows the importance in establishing a PHC network in small municipalities. According to the health professionals of SAMONTE, the profile of refereed patients changed after LIACC. The implementation of macroprocess, mainly the individual risk stratification, empowered FHT to screen patients to receive treatment at different levels of care, since this strategy was not implemented in SAMONTE before LIACC. The coverage of individual risk stratification increased from zero to 35% and 28% for patients with diabetes and hypertension, respectively. As expected, the risk stratification is usually gradually implemented, since this process depends on diagnostic tests. The increase in the prevalence of the self-reported diabetes from 4.53% in 2012 to 9.44% in 2014 also corroborates that FHT became more aware of the population’s risk conditions.

The third lesson reinforces the role of CHA and of the health managers of HU as fundamental actors in the PHC network. CHA are responsible for the visit and registry of the households, as well as for the implementation of the risk classification and stratification. The coverage of CHA visits was already universal for all target groups before LIACC, but these macroprocesses were not fully achieved. The individual risk classification had never been implemented before LIACC, while the family risk classification was carried out only once, but it was not regularly monitored and updated. The health managers, introduced by LIACC, are in charge of monitoring CHA to ensure the full implementation of these macroprocesses.

Despite these results, the implementation of LIACC posed some challenges that need to be overcome in future experiences. At first, the SAMONTE experience did not consider reorganizing the supply of diagnostic tests. This issue is important to be addressed, since LIACC did not succeed in providing universal coverage of preventive tests defined by the protocols for patients with diabetes and hypertension. Besides, as the Brazilian Health System is mixed, some individuals have dual access to healthcare services. For instance, they can visit a doctor in the private sector and receive the diagnostic tests from the public sector. This dual system allows individuals to bypass the procedures established in the public PHC network and prevent the adequate management of the supply of diagnostic tests.

Another challenge is the use and implementation of an electronic system in small municipalities. This tool is important to improve the quality of clinical records, to allow the integration of all health care units (PHC and specialized units), and to ensure the management of a longitudinal care. In SAMONTE, the electronic system should have been introduced with LIACC. However, this macroprocess was not implemented because it involves decisions and resources at Federal and State levels. Even though the computers were provided by State Health Secretary during LIACC, their use requires the development of a suitable software, the training of health professionals and adequate internet connection.

Finally, SAMONTE is a pilot experience and the scale up of ACCM is not trivial. SAMONTE was chosen to receive this intervention due to the presence of a well-organized secondary facility that has already provided a good care to the target groups compared with other municipalities. Besides, LIACC is a complex intervention. It comprises the incorporation of different tools in health care management, which involves all health professionals and their ability to understand this new management of chronic conditions. In SAMONTE, this caveat became evident when some of the health managers were dismissed due to labor regulation of the public sector in Brazil.

We believe this study is the first opportunity to address the implementation of an ACCM in a small municipality in Brazil, contributing thus to future experiences. The increase in the prevalence of chronic conditions is a reality that should be faced by low- and middle-income countries that are still consolidating a healthcare system able to tackle population needs. The PHC network is essential to provide an adequate and longitudinal care, considering the population’s aging scenario.
